# Late Results of Cox Maze III Procedure in Patients with Atrial Fibrillation Associated with Structural Heart Disease

**DOI:** 10.5935/abc.20170082

**Published:** 2017-07

**Authors:** Gustavo Gir Gomes, Wagner Luis Gali, Alvaro Valentim Lima Sarabanda, Claudio Ribeiro da Cunha, Iruena Moraes Kessler, Fernando Antibas Atik

**Affiliations:** 1 Instituto de Cardiologia do Distrito Federal - Fundação Universitária de Cardiologia (FUC), Brasília, DF - Brazil; 2 Universidade de Brasília (UnB), Brasília, DF - Brazil

**Keywords:** Atrial Fibrillation/surgery, Arrhythmias,Cardiac, Mitral Valve, Rheumatic Fever

## Abstract

**Background:**

Cox-Maze III procedure is one of the surgical techniques used in the surgical treatment of atrial fibrillation (AF).

**Objectives:**

To determine late results of Cox-Maze III in terms of maintenance of sinus rhythm, and mortality and stroke rates.

**Methods:**

Between January 2006 and January 2013, 93 patients were submitted to the cut-and-sew Cox-Maze III procedure in combination with structural heart disease repair. Heart rhythm was determined by 24-hour Holter monitoring. Procedural success rates were determined by longitudinal methods and recurrence predictors by multivariate Cox regression models.

**Results:**

Thirteen patients that obtained hospital discharge alive were excluded due to lost follow-up. The remaining 80 patients were aged 49.9 ± 12 years and 47 (58.7%) of them were female. Involvement of mitral valve and rheumatic heart disease were found in 67 (83.7%) and 63 (78.7%) patients, respectively. Seventy patients (87.5%) had persistent or long-standing persistent AF. Mean follow-up with Holter monitoring was 27.5 months. There were no hospital deaths. Sinus rhythm maintenance rates were 88%, 85.1% and 80.6% at 6 months, 24 months and 36 months, respectively. Predictors of late recurrence of AF were female gender (HR 3.52; 95% CI 1.21-10.25; p = 0.02), coronary artery disease (HR 4.73 95% CI 1.37-16.36; p = 0.01) and greater left atrium diameter (HR 1.05; 95% CI 1.01-1.09; p = 0.02). Actuarial survival was 98.5% at 12, 24 and 48 months and actuarial freedom from stroke was 100%, 100% and 97.5% in the same time frames.

**Conclusions:**

The Cox-Maze III procedure, in our experience, is efficacious for sinus rhythm maintenance, with very low late mortality and stroke rates.

## Introduction

Atrial fibrillation (AF) is the most common sustained arrhythmia in adults, with a close relationship with aging. The prevalence of AF increases from 0.2% in individuals aged 45-54 years to 8% in those aged over 75 years.^1^ Among cardiac surgery patients, AF is found in up to 50% of patients undergoing mitral valve surgery and in 1-6% of patients undergoing myocardial revascularization or aortic valve replacement.^2^

Surgical treatment of AF is an alternative, efficient therapeutic approach for long-term maintenance of sinus rhythm. The third version of Cox-Maze procedure (CM III), or traditional Maze, is considered the gold standard surgical procedure for AF.^3^ A set of incisions and sutures causes anatomical and functional changes in the atria, allowing the conduction of stimulus from the sinus node to the atrioventricular node and, concomitantly, preventing both maintenance^3^ and initiation of AF.^4,5^

CM III procedure leads to high rates of sinus rhythm maintenance^3^ and low incidence of late stroke, particularly due to closure of the left atrial appendage.^6^ On the other hand, advanced age, increased left atrial dimensions, and long-standing persistent AF have been identified as predictive factors of recurrent AF after the Maze procedure.

Due to technical complexity and assumed increase in morbidity, CM III is performed on a regular basis in relatively few centers nowadays. The use of alternate energy sources and surgical ablation procedures has simplified the traditional procedure, and been increasingly performed worldwide.^7^

The aim of the present study was to assess long-term results of CM III regarding maintenance of sinus rhythm, risk factors for recurrent arrhythmia, late mortality and survival rate free of stroke.

## Methods

This study evaluated a cohort of patients with structural heart disease-related AF, who underwent combined CM procedure from January 2006 to January 2013. Surgical treatment consisted of CM III, which was performed as described by Cox et al.^3^

The study was approved by the Ethics Committee and registered at *Plataforma Brasil* (identification number 20301113.0.0000.0026). Informed consent was obtained from all volunteers and/or caregivers who agreed to participate in the study.

Surgical indications were established based on clinical and surgical criteria, following the Brazilian Society of Cardiology guidelines. Decisions for surgery were made by the same staff, based on the risks of procedure, familiarity and previous experience with the technique, and potential benefits in each case.

Demographical and clinical data, and complementary tests were obtained retrospectively from patients' medical records. Similarly, operative characteristics and post-operative information were collected from electronic and nursing records of the procedure. All data were stored electronically and protected against unauthorized access.

Patients with more than three months of follow-up were invited to participate in the study, by attending a clinical visit with the main investigator (cardiologist) for assessment of cardiac rhythm by 12-lead electrocardiogram and 24-hour Holter monitoring. It is worth pointing out that patients continued their postoperative outpatient treatment provided by the medical staff, and continuation of antiarrhythmic drug therapy was left to the cardiologist's discretion. Occurrence of any recurrent arrhythmia episode was recorded for analysis.

Late AF recurrence was defined according to American cardiology societies' guidelines.^8^ Recurrence was defined as the occurrence of AF, atrial flutter, or atrial tachycardia lasting ≥ 30 seconds after a period of at least 3 months surgery. Episodes of early recurrent AF (AF occurring in the period from the day of surgery to the day of hospital discharge) were also verified for prediction of late recurrence.

For assessment of CMIII-related late morbidity, we evaluated the incidence of stroke and mortality.

### Surgical technique

All surgeries were performed by the same staff and using the same standard technique.

Surgery began with CMIII procedure, in which a cut was performed into the atrial walls with scissors or electrocautery, followed by continuous suture with polypropylene. Then, resection of left atrial appendage and complete isolation of pulmonary veins were performed, with an ablation line directed toward the mitral valve annulus, an ablation line to the cavo-tricuspid isthmus, a communicating incision from superior to inferior vena cava, and resection of right atrial appendage. After that, if indicated, additional procedures, concomitant to CM III were performed. Patients were then transferred to the post-operative intensive care unit.

### Statistical analysis

Categorical variables were expressed as frequencies and percentages. Normally distributed continuous variables were expressed as mean and standard-deviation. Continuous variables with non-normal distribution were expressed as median and interquartile ranges.

Efficacy of surgical treatment was assessed by the rate of maintenance of sinus rhythm at 6, 24 and 36 months. Survival rates free of stroke and death were estimate by Kaplan-Meier curves.

Univariate Cox regression analysis was used for demographic and clinical variables in the assessment of predictors of late AF recurrence. Variables with p < 0.25 in the uniariate analyses were included in the multivariate Cox regression analysis. The final multivariate logistic regression model was constructed by excluding the variables from the initial multivariate model, according to their relative importance (which was estimated by the similarity ratio test). The level of significance was set at 0.05, and analyses were performed using the SAS software, version 9.3.

Left atrial diameter was compared with late AF recurrence, and sensitivity and specificity indicators were calculated for the cutoff points and ROC curve. The chi-square test was used to assess the association between early AF recurrence and late recurrence, and to evaluate the impact of the use of arrhythmic drugs on late recurrence.

## Results

Ninety-three patients underwent surgical treatment for AF combined with correction of structural heart disease between January 2006 and January 2013. Eighty patients met the inclusion criteria. Thirteen patients who survived the perioperative period were excluded, due to loss to follow up. Mean postoperative follow-up period was 27.5 months (3-89 months).

Baseline (pre-operative) demographic, clinical and echocardiographic data are described in Tables 1, 2 and 3, respectively. Patients were of moderately advanced age (mean of 50 years), and 57 (58.8%) were women. Persistent AF and long-standing persistent AF were the most prevalent conditions, found in 70 (87.5%) patients.

**Table 1:** Demographic characteristics of patients

Characteristics	N = 80 patients	%
Male sex	33	41.25%
Age*	49.94 ±12.06*	
Duration of atrial fibrillation (months)†	15 (8-36)†	
Paroxystic atrial fibrillation	10	12.5%
Persistent atrial fibrillation	23	28.75%
Long-standing persistent atrial fibrillation	47	58.75%

* Values indicate Mean (Standard Deviation);

† Values indicate median and interquartile range.

**Table 2:** Clinical characteristics of patients in the preoperative period

Characteristics	N = 80 patients	%
Arterial hypertension	34	42.5%
Diabetes mellitus	6	7.5%
Coronary artery disease	10	12.5%
History of stroke/TIA*	10	12.5%
Valve disease	75	93.75%
Rheumatic valve disease	63	78.75%
Congenital heart disease	4	5%
History of cardiac surgery	11	13.75
Medications		
Warfarin	49	61.25%
Amiodarone	21	26.25%
ACEIs/ARBs†	53	66.25%
Beta-blockers	61	76.25%

* TIA: transient ischemic accident;

† ACEI: angiotensin converting enzyme inhibitors; ARBs: angiotensin II receptor blockers.

**Table 3:** Echocardiographic characteristics of patients in the preoperative period

Echocardiography	N = 80 patients	%
Ejection fraction (%)*	61(56-66)*	
LA diameter (mm)	55.66±9.41†	
LA volume index (mm/m^2^)‡	61(44-94)*	
LV diastolic diameter (mm)	51(45,5-60,5)*	
LV systolic diameter (mm)	35(30-43)*	
Tricuspid regurgitation degree§	Mild	64%
	Moderate	29%
	Increased	7%
PASP (mmHg) /(n = 74)//	47.5(40-60)*	

* Values indicate Median

† Values indicate Mean (standard deviation);

‡ Left atrial index volume: data obtained for 54 patients;

§ Degree of regurgitation of the tricuspid valve: data obtained for 79 patients;

// PASP: pulmonary artery systolic pressure - data obtained for 74 patients. LA: left atrium; LV: left ventricle.

Heart valve disease was identified in 75 (93.8%), and rheumatic mitral valve disease was the most prevalent one, found in 63 (78.8%) patients. Coronary heart disease was diagnosed in 10 (12.5%) patients. There was a considerable increase (mean of 55 mm) in mean left atrial diameter.

Table 4 shows patients' operative data, including the type of surgery performed in combination with the CM III procedure. Sixty-seven (83.7%) patients underwent treatment of mitral valve, either alone or combined with other valve repair procedures. Myocardial revascularization (alone or in combination with valve repair) was found in 6 (7.5%) patients. Five patients underwent atrial septal repair by closure of the interatrial communication (6.3%).

**Table 4:** Operative data of patients

COX-maze III-combined surgery	N = 80 patients	%
Mitral alone	29	36.25%
Mitral + tricuspid	27	33.75%
MRS alone †	2	2.5%
Mitral + MRS	3	3.75%
Aortic alone	2	2.5%
Aortic + MRS	1	1.25%
Mitral + aortic	3	3.75%
Mitral + aortic + tricuspid	5	6.25%
Others (including congenital	8	10%
Extracorporeal time (minutes) ‡	130 (117-150)*	
Time at postoperative ICU (days) (n = 78)	3 (3-5)*	

* Median and interquartile range;

† MRS: myocardial revascularization surgery;

‡ Extracorporeal time; ICU: intensive care unit.

### Perioperative results

During hospitalization (perioperative period), 32 (40%) patients had early recurrent AF, atrial flutter or atrial tachycardia. Sixteen patients (20%) had bradyarrhythmias, including atrioventricular block, sinus node dysfunction, sinus bradycardia, and junctional rhythm. Three (3.8%) patients required permanent pacemaker implantation. No patient died in the perioperative period.

On the day of discharge, 66 patients (82.5%) showed sinus rhythm and 14 patients (17.5%) did not. Three (3.8%) were using a pacemaker, and 13 (16.3%) had AF, atrial flutter or atrial tachycardia.

### Clinical follow-up after hospital discharge

Rates of sinus rhythm maintenance at 6, 24 and 36 months were 88%, 85.1% and 80.6%, respectively. The number of patients who underwent Holter monitoring and electrocardiogram at these time points were 76, 46 and 31, respectively.

According to the multivariate analysis, predictors of late AF recurrence were female sex (HR 3.52; 95% CI 1.21-10.25; p = 0.02), presence of coronary artery disease (HR 4.73; 95% CI 1.37-16.36; p = 0.01) and increased left atrial diameter (HR 1.05; 95% CI 1.01-1.09; p = 0.02). For every one millimeter increase in left atrial diameter, AF recurrence increased by 5% (Table 5).

**Table 5:** Predictors of late recurrence of atrial fibrillation – crude hazard ratio and adjusted odds ratio for arrhythmia recurrence, by selected demographical and clinical variables

	Hazard ratio – HR (95% CI)			
	Crude	p value	Adjusted	p value*
Sex		0.0633		0.0209
Male	1		1	
Female	2.60 (0.95 – 7.13)	0.0633	3.52 (1.21 – 10.25)	0.0209
Amiodarone		0.0892		
No	3.54 (0.82 – 15.24)	0.0892		
Yes	1			
Creatinine	0.14 (0.02 – 0.91)	0.0392		
Coronary artery disease		0.1852		0.0142
No	1		1	
Yes	2.11 (0.70 -6.38)	0.1852	4.73 (1.37 – 16.36)	0.0142
Left atrial diameter	1.04 (0.99 – 1.08)	0.0967	1.05 (1.01 – 1.09)	0.0256

Late AF recurrence was found in 20.5% of patients with left atrial diameter ≤ 56 mm, and in 34.3% of those with left atrial diameter > 56 mm. Area under the ROC curve was 0.62 (95%CI 0.48-0.75), with sensitivity of 57% and specificity of 40%.

The impact of early recurrence of AF on late recurrence was evaluated by using the chi-square test (Table 6). No correlation between early and late AF recurrence was observed.

**Table 6:** Impact of early recurrence of atrial fibrillation on late recurrence of the disease

Late recurrence	Early recurrence (%)		p value
	Absent	Present	0.4066
Absent	37 (77.08)	22 (68.75)	
Present	11 (22.92)	10 (31.25)	
Total	48 (60.00)	32 (40.00)	

* chi-squared test.

The effect of the use of antiarrhythmic drugs (amiodarone) on late recurrence of AF was analyzed from data of 78 patients (97.5% of total). Twenty-one patients used amiodarone at long-term follow-up; 10 (48%) of them without recurrent AF and 11 (52%) with recurrent AF. The chi-square test revealed an inverse relationship between the use of amiodarone and recurrence of arrhythmia, indicating that there was no protective effect of this medication on late arrhythmia recurrence (Table 7).

**Table 7:** Association between antiarrhythmics and atrial fibrillation recurrence

Use of antiarrhythmics*	Recurrence of AF (%)		Total
	No	Yes	
No	47 (82.46)	10 (17.54)	57
Yes	10 (47.62)	11 (52.38)	21

* Chi-squared test = 9.47; p = 0.0021. AF: atrial fibrillation.

### Mortality, incidence of stroke, and need for permanent pacemaker implant at late follow-up of CMIII

At long-term follow-up, one (1.25%) death was recorded due to complications of mitral valve surgical repair eight months after the CMIII procedure. Actuarial survival at 12, 24 and 48 months was 98.5%, and the number of exposed patients at these time points was 66, 47 and 18, respectively. Survival rates free of stroke at 12, 24 and 48 months were 100%, 100% and 97.5%. A total of 59 (75.0%) patients used oral anticoagulation at late follow-up, 44 (56.0%) because of prosthetic heart valve.

No patient required implantation of permanent pacemaker at late follow-up.

## Discussion

The present study aimed to assess long-term results of the "cut-and-sew" CM III technique in a cohort of patients with AF associated with structural cardiac disease, in terms of maintenance of sinus rhythm, morbidity and mortality, and to determine possible predictors of late AF recurrence.

### Success rate of maintenance of sinus rhythm and predictive factors of late recurrence of AF in patients undergoing CM III

Meaningful results were reported by several groups performing the CM III procedure in long-term maintenance of sinus rhythm, due to the consistent nature of the lesion in the atriums and the guarantee of obtaining transmural lesions by this technique.^9^

Stulak et al.^9^ reported their experience with the surgical treatment of 1,540 patients with AF at Mayo Clinic, 514 of them undergoing the CM III procedure. In a median follow-up period of 34 months (maximum of 18.5 years), 80% of patients were free from AF and without antiarrhythmic medications. CMIII was superior in maintenance of sinus rhythm as compared with other surgical approaches for AF treatment.

Kamata et al.^10^ were one of the first authors to investigate the predictors of AF recurrence in the late postoperative period of CMIII. The authors demonstrated that a low "f" wave amplitude (<1mm) and left atrial diameter > 65 mm were inversely related with late sinus rhythm restoration.

In 2005, Gaynor et al.,^11^ in a study evaluating the predictors of late AF recurrence after CM surgery, in a mean follow-up of 6 years, demonstrated that a longer duration of preoperative AF was correlated with higher incidence of late AF. In addition, CM III procedure achieved higher success rates compared with other versions of the CM procedure. Left atrial dimensions were not investigated in this study.

Left atrial diameter as a predictor of late AF recurrence after CM surgery was analyzed in a meta-analysis by Sunderland et al.^12^ A diameter > 60 mm showed a sensitivity of 100% for AF recurrence, whereas as a diameter < 48.3 mm showed a sensitivity of 100% for reversal of the sinus rhythm. In the study by Gillinov et al.^13^ performed at the Cleveland Clinic, the following predictors of AF recurrence after combined CM and mitral valve surgery were reported: longer duration of preoperative AF, larger left atrial diameter, older age, and higher left ventricular mass index. In agreement with these reports, our findings demonstrated that left atrial diameter was an independent predictor of late AF recurrence. However, in contrast with the literature, duration of preoperative AF and the type of AF were not associated with higher postoperative AF recurrence. This may be explained by the small number of patients.

Increased left atrial dimensions and AF with longer duration lead to greater degree of electrical and mechanical remodeling of the left atrium. According to Kottkamp,^14^ the presence of interstitial fibrosis would lead to abnormal conduction and activation of atrial electrical impulse, and increased risk for AF.

In our study, the presence of coronary artery disease was considered a predictive factor of AF recurrence, which may be analyzed under two aspects: first, this data corroborates previous findings suggesting that AF physiopathology in coronary patients is correlated with more advanced stages of myocardial dissease.^15,16^ In this case, one may presume that these patients would be more likely to AF recurrence due to the presence of interstitial fibrosis in atrial tissue.

In contrast, in 2003, Damiano et al.^15^ already showed excellent results of myocardial revascularization combined with CM III procedure in late efficacy and low mortality index in a group of 47 coronary patients. Other studies have demonstrated this favorable trend of AF surgical repair in coronary disease patients. In fact, current American^17^ and Brazilian^18^ cardiology societies' guidelines include all types of structural heart diseases as indications for combined treatment of AF by surgical approach.

In our study, female sex was significantly correlated with higher late AF recurrence, which was a distinct characteristic of our study group; or, rather, it may be resulted from the small size of the sample. Further studies are needed to draw conclusions of this relationship between sex and AF recurrence.

We did not find any correlation between early recurrence of AF and late recurrence of arrhythmia. There were 32 cases (40%) of early recurrence of atrial tachyarrhythmias. Other studies, such as those by Gaynor et al.^11^ and Gillinov et al.,^13^ reported 44% and 38% of early recurrence, respectively. In both studies, there was a high successful rate of sinus rhythm maintenance at long-term.

Up to 3 months after surgical treatment of AF, the substrates and triggers of recurrent atrial tachyarrhythmias may be different from the determinants of baseline arrhythmia and, for this reason recurrences within this period may not be considered a therapeutic failure *per se*.^19^

The presence or not of predictors of late AF recurrence is a valuable information to guide the surgical treatment of AF, especially in those patients with indications of combined surgical heart repair. In this context, back in 1999, Kalil et al^20^ proposed that the Maze procedure should be performed in all patients with long-standing, persistent AF, undergoing mitral valve surgery.

According to Pinho-Gomes et al.,^21^ surgical repair of AF in patients with rheumatic valve disease would have inferior results, due to the presence of fibrosis and more severe inflammation in these patients. However, this was not observed in the our study, although 78.8% of the patients had rheumatic valve disease. Albrecht et al.,^22^ also investigating a group of patients predominantly composed of rheumatic patients undergoing two different surgeries, one of them modified CMIII, also achieved high success rates in the maintenance of sinus rhythm. Also corroborating with our findings, in the study by Abreu-Filho et al.,^23^ 70 patients with rheumatic mitral valve disease and long-standing persistent AF were allocated to undergo mitral surgery alone or mitral surgery plus modified CM III. The results showed marked differences in sinus rhythm restoration, with favorable results for the group that underwent surgical correction of AF.

High success rates in sinus rhythm maintenance reported in the main centers may be influenced by insufficient monitoring of cardiac rhythm. In general, the longer the period of electrocardiographic monitoring, the higher the chance of AF recurrence, usually asymptomatic.^24^ Based on American and European arrhythmia societies' recommendations,^8^ the most effective method to assess the cardiac rhythm is Holter monitoring for up to 7 days.

In most of studies, including in the present one, cardiac rhythm was assessed by electrocardiography in each visit and by at least one 24-hour Holter recording.

Ad et al.^24^ compared thee methods for the analysis of cardiac rhythm - electrocardiogram, 24-hour Holter monitoring and long-term (5 days) monitoring. The results revealed higher sensitivity of long-term cardiac monitoring over the other techniques.

In addition to assessing cardiac rhythm, we evaluated the possible effect of antiarrhythmic drugs on AF recurrence. In our study, the use of amiodarone did not provide additional protection to CM III procedure against late AF recurrence. This finding is similar to that reported by Schuetz et al.^25^ on surgical treatment of AF using microwave energy ablation. Twelve months after surgery, the authors found no significant difference in AF recurrence between the groups treated and not treated with antiarrhythmic drugs.

### Late results of CM surgery in terms of mortality, stroke and pacemaker implantation

There was one late death (1.3%); two (2.5%) patients had ischemic stroke, and no patient required pacemaker implantation.

Due to technical complexity of CM III, the surgery is not performed in some institutions, which may contribute to increased mortality and morbidity rates. The CM IV procedure has been proposed to simplify the original CM III. Weimer et al.^26^ showed similar efficacy, shorter operating times and lower complication of CM IV compared with CM III. However, in a population of 212 patients, no difference in 30-day mortality was found between CM III and CM IV, despite the higher frequency of perioperative complications in the CM III group.

Combination of surgical treatment of AF with cardiac surgery causes a minimal increase in mortality and morbidity, with a mortality rate of 4%, in comparison with the rate of 3.3% of cardiac surgery alone.^27^

Although we did not have a control group composed of patients undergoing cardiac surgery alone, the occurrence of one death (1.3%) at long-term follow-up corroborates the literature in the sense that there was no significant effect of CM III on mortality.

An important issue to be discussed is the need of oral anticoagulation in preventing thromboembolic events, taking into consideration restoration and maintenance of sinus rhythm in the postoperative period of CM III. Dr. Cox himself has reported that the technique proposed by his group reduced the incidence of stroke in the perioperative period to less than 1%, and practically eliminated the risk of late stroke.^6^

In our study, the incidence of late stroke of 2.5% is in agreement with the reports of low incidence of cerebrovascular events. However, so far, there has been no strong evidence for interruption of oral anticoagulation in the postoperative period of surgical repair of AF, regardless of maintenance of sinus rhythm.^19^ Decision on the suspension of anticoagulation should be individualized.

Another important issue related to CM III surgery is the higher requirement for pacemaker implantation for bradyarrhythmias. According to Gillinov et al.,^28^ this may occur in 5-20% of cases. In our study, there were 3 patients (3.8%) in this condition. This may be explained not only by biatrial lesions performed during surgery, but also by an underlying sinus node dysfunction in many patients.^21^

### Limitations

The present study has some limitations to be considered. One of them is the retrospective nature of the study, in which all patients who had submitted to CM III in the study period were included in the study. In addition, there was no control group for comparisons of cardiac rhythm in the postoperative period.

Second, assessment of cardiac rhythm was performed only by electrocardiogram and 24-hour Holter monitoring. We did not use other more sensitive methods for detection of AF recurrence, including Holter recordings for up to seven-day, and non-implantable and implantable cardiac monitors available at the market. In addition, success rates may have been overestimated in sinus rhythm maintenance.

Third, we did not analyze any parameter of left atrial contractility after AF surgical repair. It is known that, despite sinus rhythm recovery, a group of patients does not show any improvement of left atrial contractile function, which may result from scars and electrical isolation areas created during the procedure.^19^

Finally, although the low incidence of cerebrovascular events in the late post-operative period in our study was in agreement with previous studies, the possibility that it may have been influenced by the high prevalence of warfarin use (75% of patients) cannot be ruled out.

## Conclusions

Information on the late results of CM III with respect to sinus rhythm maintenance and late recurrence of AF contributes to proper indication of the surgery combined with structural heart disease repair. In our population, success rates of late outcomes of the surgery were comparable with those of major centers. In addition, low mortality index and low incidence of stroke were achieved. On the other hand, increased left atrial diameter, coronary heart disease and female sex were shown to be suboptimal predictors of CM III surgery outcomes. The association between sex and CM III outcome needs to be confirmed by further studies.

## References

[1] Davis RC, Hobbs FD, Kenkre JE, Roalfe AK, Ilês R, Lip GY (2012). Prevalence of atrial fibrillation in the general population and
in high-risk groups: the ECHOES study. Europace.

[2] Ministério da Saúde (2009). Parecer Técnico-Científico: Sistema de
ablação por radiofreqüência no tratamento
cirúrgico da fibrilação atrial.

[3] Cox JL, Schuessler RB, D'agostino HJ Jr, Stone CM, Chang BC, Cain ME (1991). The surgical treatment of atrial fibrillation, III: development
of a definitive surgical procedure. J Thorac Cardiovasc Surg.

[4] Haissaguerre M, Jais P, Shah DC, Takahashi A, Hocini M, Quiniou G (1998). Spontaneous initiation of atrial fibrillation by ectopic beats
originating in the pulmonary veins. N Engl J Med.

[5] Prasad SM, Maniar HS, Camillo CJ, Schuessler RB, Boineau JP, Sundt 3rd TM (2003). The Cox maze III procedure for atrial fibrillation: long-term
efficacy in patients undergoing lone versus concomitant
procedures. J Thorac Cardiovasc Surg.

[6] Cox JL (2014). A brief overview of surgery for atrial
fibrillation. Ann Cardiothorac Surg.

[7] Choi JB, Park HK, Kim KH, Kim MH, Kuh JH, Lee MK (2013). Predictive factors of sustained sinus rhythm and recurrent atrial
fibrillation after the maze procedure. Korean J Thorac Cardiovasc Surg.

[8] Calkins H, Brugada J, Packer DL, Cappato R, Chen SA, Crijns HJ, European Heart Rhythm Association (EHRA), European Cardiac Arrhythmia Scoiety (ECAS), American College of Cardiology (ACC), American Heart Association (AHA), Society of Thoracic Surgeons (STS) (2007). HRS/EHRA/ECAS expert Consensus Statement on catheter and surgical
ablation of atrial fibrillation: recommendations for personnel, policy,
procedures and follow-up. A report of the Heart Rhythm Society (HRS) Task
Force on catheter and surgical ablation of atrial
fibrillation. Heart Rhythm.

[9] Stulak JM, Suri RM, Burkhart HM, Daly RC, Dearani JA, Greason KL (2014). Surgical ablation for atrial fibrillation for two decades: are
the results of new techniques equivalent to the Cox maze III
procedure?. J Thorac Cardiovasc Surg.

[10] Kamata J, Kawazoe K, Izumoto H, Kitahara H, Shiina Y, Sato Y (1997). Predictors of sinus rhythm restoration after Cox maze procedure
concomitant with other cardiac operations. Ann Thorac Surg.

[11] Gaynor SL, Schuessler RB, Bailey MS, Ishii Y, Boineau JP, Gleva MJ (2005). Surgical treatment of atrial fibrillation: predictors of late
recurrence. J Thorac Cardiovasc Surg.

[12] Sunderland N, Maruthappu M, Nagendran M (2011). What size of left atrium significantly impairs the success of
maze surgery for atrial fibrillation. Interact Cardiovasc Thorac Surg.

[13] Gillinov AM, Sirak J, Blackstone EH, McCarthy PM, Rajeswaran J, Pettersson G (2005). The Cox maze procedure in mitral valve disease: predictors of
recurrent atrial fibrillation. J Thorac Cardiovasc Surg.

[14] Kottkamp H (2013). Human atrial fibrillation substrate: towards a specific fibrotic
atrial cardiomyopathy. Eur Heart J.

[15] Damiano RJ Jr, Gaynor SL, Bailey M, Prasad S, Cox JL, Boineau JP (2003). The long-term outcome of patients with coronary disease and
atrial fibrillation undergoing the Cox maze procedures. J Thorac Cardiovasc Surg.

[16] Melo J, Santiago T, Aguiar C, Berglin E, Knaut M, Alfieri O (2008). Surgery for atrial fibrillation in patients with mitral valve
disease: Results at five years from the International Registry of Atrial
Fibrillation Surgery. J Thorac Cardiovasc Surg.

[17] January CT, Wann LS, Alpert JS, Calkins H, Cigarroa JE, Cleveland JC Jr, American College of Cardiology, American Heart Association Task Force on Practice
Guidelines (2014). 2014 AHA/ACC/HRS guideline for the management of patients with
atrial fibrillation: a report of the American College of Cardiology/American
Heart Association Task Force on Practice Guidelines and the Heart Rhythm
Society. J Am Coll Cardiol.

[18] Magalhães LP, Figueiredo MJ, Cintra FD, Saad EB, Kuniyoshi RR, Teixeira RA (2016). II Diretrizes Brasileiras de fibrilação
atrial. Arq Bras Cardiol.

[19] Abo-Salem E, Lockwood D, Boersma L, Deneke T, Pison L, Paone RF (2015). Surgical treatment of atrial fibrillation. J Cardiovasc Electrophysiol.

[20] Kalil RA, Maratia CB, D'Avila A, Ludwig FB (1999). Predictive factors for persistence of atrial fibrillation after
mitral valve operation. Ann Thorac Surg.

[21] Pinho-Gomes AC, Amorim MJ, Oliveira SM, Leite-Moreira AF (2014). Surgical treatment of atrial fibrillation: an updated
review. Eur J Cardiothorac Surg.

[22] Albrecht A, Kalil RA, Schuch L, Abrahão R, Sant'Anna JR, de Lima G (2009). Randomized study of surgical isolation of the pulmonary veins for
correction of permanent atrial fibrillation associated with mitral valve
disease. J Thorac Cardiovasc Surg.

[23] Abreu-Filho CA, Lisboa LA, Dallan LA, Spina GS, Grinberg M, Scanavacca M (2005). Effectiveness of the maze procedure using cooled-tip
radiofrequency ablation in patients with permanent atrial fibrillation and
rheumatic mitral valve disease. Circulation.

[24] Ad N, Henry L, Hunt S, Barnett S, Stone L (2009). The Cox Maze III procedure success rate: comparison by
electrocardiogram, 24-hour holter monitoring and long-term
monitoring. Ann Thorac Surg.

[25] Schuetz A, Schulze CJ, Sarvanakis KK, Mair H, Plazer H, Kilger E (2003). Surgical treatment of atrial fibrillation using microwave energy
ablation: a prospective randomized clinical trial. Eur J Cardiothorac Surg.

[26] Weimar T, Schena S, Bailey MS, Maniar HS, Schuessler RB, Cox JL (2012). The cox-maze procedure for lone atrial fibrillation: a
single-center experience over 2 decades. Circ Arrhythm Electrophysiol.

[27] Kong MH, Lopes RD, Piccini JP, Hasselblad V, Bahnson TD, Al-Khatib SM (2010). Surgical Maze procedure as a treatment for atrial fibrillation: a
meta-analysis of randomized controlled trials. Cardiovasc Ther.

[28] Gillinov AM, Blackstone EH, McCarthy PM (2002). Atrial fibrillation: current surgical options and their
assessment. Ann Thorac Surg.

